# High doses of dexamethasone induce endoplasmic reticulum stress-mediated apoptosis by promoting calcium ion influx-dependent CHOP expression in osteoblasts

**DOI:** 10.1007/s11033-021-06806-y

**Published:** 2021-10-26

**Authors:** Yunshan Guo, Dingjun Hao, Huimin Hu

**Affiliations:** Department of Spinal Surgery, Hong Hui Hospital, Xi’an Jiao Tong University, Xi’an, 710054 Shaanxi People’s Republic of China

**Keywords:** Osteoporosis, Dexamethasone, Apoptosis, Endoplasmic reticulum stress, CHOP, Osteoblasts

## Abstract

**Background:**

The long-term use of dexamethasone (Dex), a well-known immunosuppressant, leads to an imbalance in bone metabolism and rapid decline of bone mineral density due to apoptosis of osteoblasts. The molecular mechanisms by which Dex induces osteoblast apoptosis remain unclear.

**Materials and methods:**

MC3T3-E1 cells were treated with 0, 10^−8^, 10^−6^, and 10^−4^ M Dex for 24 h. ATF6, phosphorylated PERK, PERK, phosphorylated IRE1, and IRE1 expression, cell apoptosis, and caspase-12 and caspase-3 activity were measured. CHOP expression and calcium ion influx rate were measured in cells treated with 0 and 10^−4^ M Dex for 24 h. The effect of 2-APB treatment was assessed in cells treated with 0 or 10^−4^ M Dex.

**Results:**

Levels of ATF6 and phosphorylated PERK and IRE1 increased in a dose-dependent manner in MC3T3-E1 cells treated with 10^−8^, 10^−6^, and 10^−4^ M Dex, compared to the control group (P < 0.05). Cells treated with 10^−6^ and 10^−4^ M Dex had significantly increased apoptotic rates and caspase-12 and caspase-3 activities (P < 0.05). Cells treated with 10^−4^ M Dex had significantly increased CHOP levels and calcium ion influx rates (P < 0.05). Combined treatment with 10^−4^ M Dex and 2-APB abrogated the observed increases in cell apoptosis and caspase-12 and caspase-3 activities (P < 0.05).

**Conclusions:**

High doses of Dex induce CHOP expression by promoting calcium ion influx-dependent induction of ATF6, phosphorylated PERK and phosphorylated IRE1, which induce endoplasmic reticulum stress-mediated apoptosis in osteoblasts. 2-APB protects the osteoblasts from the effects of Dex, preventing endoplasmic reticulum stress-mediated apoptosis.

## Introduction

Dexamethasone (Dex) is an anti-inflammatory and immunosuppressive drug that is widely used in the treatment of rheumatoid arthritis, inflammatory bowel disease, chronic obstructive pulmonary disease, and other diseases. High doses of Dex may cause glucocorticoid-induced osteoporosis (GIOP) [1–3]. In patients receiving Dex, 30–50% of patients experience a fracture, with a 75% increased risk of fracture before a significant decrease in bone mineral density (BMD) occurs within the first 3 months of Dex treatment. The decrease in BMD reaches its peak at 3 to 6 months after the initiation of Dex treatment, and then continues to decrease steadily over time [4–6]. The rapid decrease in BMD observed during the early stages of Dex treatment is caused by an increase in osteoclast activity, whereas the slower decrease in BMD over the long term is related to a decrease in the activity of osteoblasts [7, 8].

Osteoblasts play an important role in ensuring the normal bone mass of the body by forming bone tissue. Osteoporosis is a systemic metabolic change characterized by low bone mass, which can lead to increased bone fragility and increase the risk of fracture [9, 10]. Recent studies have shown that the occurrence of osteoporosis is related to endoplasmic reticulum (ER) stress [11]. The ER is an important membranous organelle that is responsible for protein synthesis and folding, regulating the balance of calcium ions inside and outside the cell, and calcium storage. When intracellular calcium ion homeostasis is unbalanced, and unfolded or misfolded proteins accumulate in the ER cavity, the intracellular environment is affected and the ER loses its normal physiological function, leading to a series of adaptive regulatory processes termed ER stress [12, 13].

Different intensities of ER stress have a bidirectional regulatory effect on osteoblast function. Under low levels of ER stress, the expression of Runx2 and other bone forming factors in osteoblasts increases, which promote the formation and reconstruction of bone tissue and effectively alleviate the progression of osteoporosis [14]. However, under excessive ER stress, the synthesis of ATF4 and ATF3 increases, promoting the expression of CHOP. High levels of CHOP activate the apoptotic pathway and accelerate the apoptosis of osteoblasts, leading to osteoporosis [15, 16]. Although both Dex and ER stress induce osteoporosis by mediating osteoblast apoptosis, whether Dex leads to osteoblast apoptosis by inducing osteoblast ER stress is not clear.

In the present study, we explored the effect of Dex on ER stress in an osteoblast cell line to identify the mechanisms by which Dex induces osteoporosis. The effects of 2-APB, which can modify calcium channels, on osteoblasts receiving Dex was assessed in parallel to determine if calcium antagonists can improve Dex-induced osteoporosis. Our findings could provide new targets for the treatment of iatrogenic osteoporosis caused by high doses of Dex.

## Materials and methods

### Cell culture

Mouse calvaria osteoblasts, MC3T3-E1, were purchased from Cell Resource Center (Shanghai Institute for Biological Sciences, Chinese Academy of Sciences, Beijing, China). MC3T3-E1 cells were cultured in α-minimum essential medium containing 10% FBS (16000-044, Gibco, USA), 100 U/mL penicillin, and 100 g/mL streptomycin under 5% CO2 in an incubator at a constant temperature of 37 °C. When the cells reached 80–90% fusion, cells in the logarithmic growth phase were selected for experimentation.

### Cell transfection

Cells were seeded in 6-well plates at a density of 2 × 105 cells/well. CHOP siRNA and control siRNA were transfected using Lipofectamine 2000 Opti Mem I (11,668,027, Invitrogen, USA) according to the manufacturer’s instructions. The CHOP siRNA sequence was 5′-GCGCATGAAGGAGAAAGAACAGG-3′, and the control siRNA sequence was 5′-TTCTCCAACTGTGACGTGT-3′. After transfection for 24 h, the culture medium was changed, and cells were cultured in an incubator at 37 °C under 5% CO2.

### Western blotting

After cells were lysed, the cell lysates was quantified using a BCA Protein Assay Kit (ab102536, Abcam, UK), diluted to the same concentration, and heated at 95 °C for 8 min with 4× sample loading buffer. Proteins were separated on an 8% acrylamide gel electrophoretically using SDS-PAGE, then transferred onto a PVDF membrane and blocked with 5% skim milk. Primary antibodies were diluted with TBST as follows: ATF6 (#65,880, Cell Signaling Technology, USA) (1:1000), phosphorylated PERK (PA5-40294, Invitrogen) (1:500), PERK (PA5-99447, Invitrogen) (1:800), phosphorylated IRE1 (PA1-16927, Invitrogen) (1:500), IRE1 (MA5-14991, Invitrogen) (1:800), CHOP (#5554, Cell Signaling Technology) (1:500), and GAPDH (A21994, Invitrogen) (1:1000). The PVDF membrane was incubated overnight with the diluted primary antibodies at 4 °C and then with horseradish peroxidase-labeled secondary antibodies, diluted at 1:20,000 in TBST, at room temperature in the dark for 1 h. Finally, protein bands were detected using the Odyssey two-color infrared laser imaging system.

### TUNEL staining

Cell apoptosis was detected using the TUNEL Assay Kit (ab66108, Abcam). Cells in suspension were added to the inner cover glass of a cell culture plate and cultured overnight as before. Cells were fixed with 4% paraformaldehyde at room temperature for 10 min. Sheep serum was dropped onto the cover glass in 50 µL aliquots and incubated for 20 min at room temperature. After washing with PBS, 50 µL of anti-TUNEL reaction mixture was added, and cells were incubated at 37 °C for 60 min. After washing with PBS, 50 µL of DAPI (at 1:100 dilution) was added and the mixture incubated at room temperature for 8 min. Cover glasses were covered with 50% glycerin solution. The proportion of TUNEL positive cells was determined using an inverted fluorescence microscope.

### Caspase-12 and caspase-3 activity assays

Caspase-12 activity was detected using the Caspase-12 Assay Kit (ab65664, Abcam). To measure caspase-12 activity, cells were removed from plates by digesting with trypsin, collected in a centrifuge tube and counted, and approximately 5 × 104 cells were removed and centrifuged. The supernatant was discarded, and 50 µL of cell lysis buffer was added, and the cells were lysed in an ice bath for 15 min. Reaction buffer (50 µL) was added to each tube and mixed on a vortex, and then 5 µL of ATAD-AFC was added to each tube, mixed, and incubated at 37 °C for 90 min. Aliquots of each reaction mixture (100 µL) were transferred to a 96-well plate, and the absorbance was measured with an excitation wavelength of 400 nm and an absorption wavelength of 505 nm on a microplate reader.

Caspase-3 activity was detected using the Caspase-3 Assay Kit (ab252897, Abcam). Caspase-3 activity was measured similarly, except that 2 × 105 cells were lysed in 100 µL of lysis buffer, and 10 µL of cell lysate was combined with 10 µL of AC-DEVD-PNA and 80 µL of detection buffer in wells of a 96-well plate. The absorbance was also measured on a microplate reader, with an excitation wavelength of 400 nm and an absorption wavelength of 505 nm.

### Calcium ion influx detection

Cells were cultured overnight as before and allowed to adhere, after which they were washed twice with PBS. After the supernatant was discarded, 5 M Fluo-8 AM solution, a calcium ion-binding dye (ab142773, Abcam) was added to the plate and incubated for 60 min in the dark at room temperature. The supernatant was removed and discarded, and 4 M TG solution was added to the plate and incubated for 40 min in the dark at room temperature. After the supernatant was discarded, cells were washed twice in calcium ion-free PBS. The fluorescent signal was measured using a confocal laser microscope. The emission wavelength was 510 nm, and the excitation wavelengths were 380 nm/340 nm. After the initial fluorescence reading was obtained for 30 s, 2 mM CaCl2 solution was added to each well, and the fluorescent signal was measured again to determine the rate of influx of calcium ions.

### Real-time PCR

Total RNA was extracted using the TRIzol reagent (12,183,555, Invitrogen) and reverse-transcribed to cDNA using the SYBR PrimeScriptTM RT-PCR Kit II (RR086A, Takara Bio, Japan), according to the manufacturers’ instructions. GAPDH was used as the reference gene. The forward primer for CHOP was 5′-GACGCTTCACTACTCTTGACCCTGCG-3′, and the reverse primer was 5′-GGATGTGCGTGTGACCTCTGT-3′. The forward primer for GAPDH was 5′-CGCTCTCTGCTCCTCCTGTT-3′, and the reverse primer was 5′- CCATGGTGTCTGAGCGATGT-3′. The PCR conditions were as follows: pre-denaturation at 95 °C for 5 min, followed by 37 cycles of 94 °C denaturation for 20 s, annealing at 59 °C for 20 s, and elongation at 72 °C for 20 s. Ct values were used to quantify the relative transcription levels of CHOP mRNA. The mRNA relative expression level was calculated using the 2^−∆∆Ct^ method.

### Statistical analysis

All statistical analysis was performed using SPSS 22.0 statistical software (IBM, USA). All experiments were independently replicated 3–6 times, and data are expressed as the mean ± standard deviation. The data from each group was normally distributed and had homogeneous variance. One-way ANOVA was used for comparisons between multiple groups, and an LSD-t test was used for pairwise comparisons. A P < 0.05 was considered to be statistically significant.

## Results

### High doses of Dex induce ER stress-mediated apoptosis in osteoblasts

**Fig. 1:**
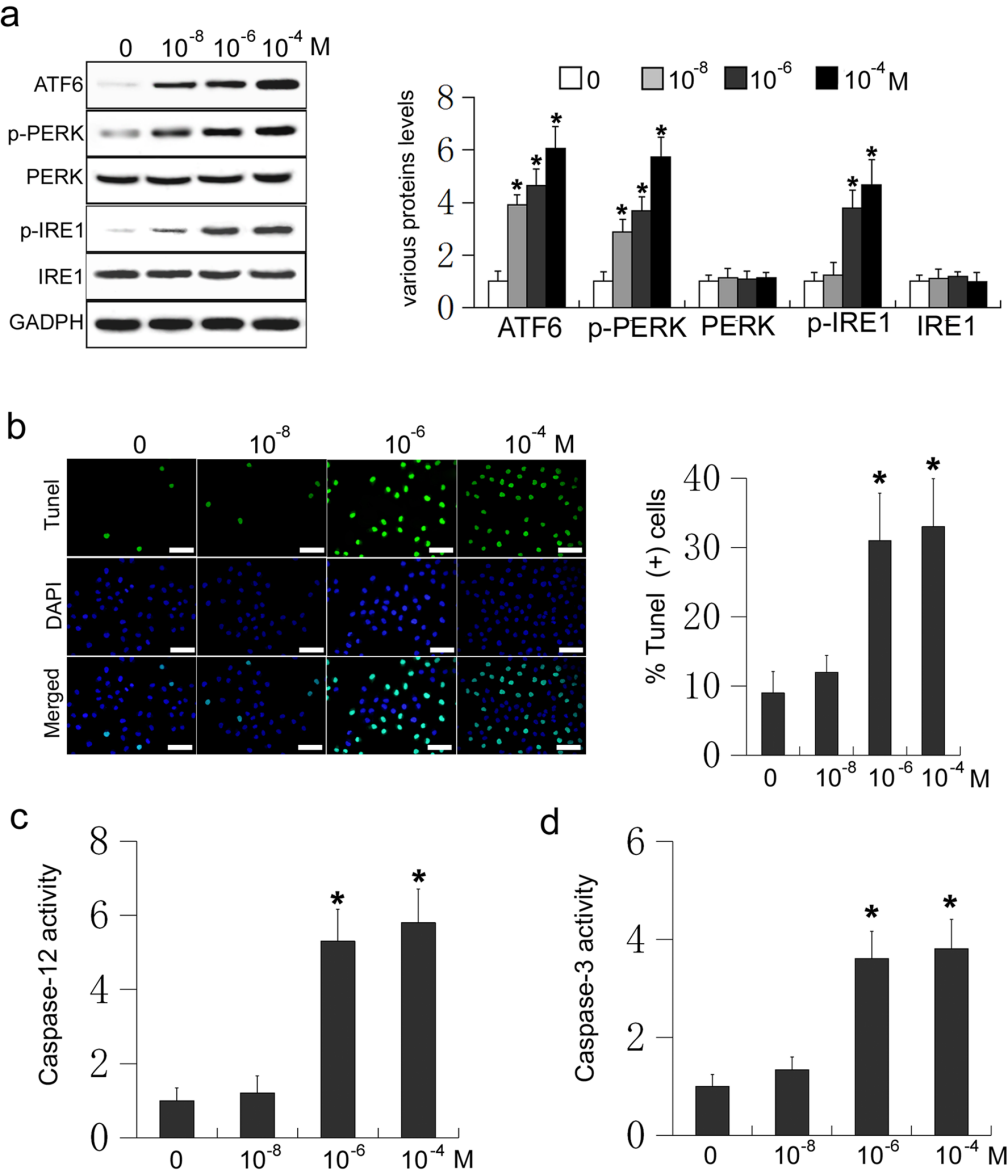
MC3T3-E1 cells were treated with Dex (0, 10^−8^, 10^−6^, and 10^−4^ M) for 24 h. **a** Western blot of ATF6, phosphorylated PERK (p-PERK), PERK, phosphorylated IRE1 (p-IRE1), and IRE1 in MC3T3-E1 cells. **b** TUNEL staining was used to detect the effect of different concentrations of Dex on apoptosis in MC3T3-E1 cells (Scale=50 μm); **c** Caspase-12 activity was measured in cells receiving Dex; **d** Caspase-3 activity was measured in cells receiving Dex

ER stress-induced osteoblasts apoptosis is one of the pathogenic features of osteoporosis [11]. To determine whether Dex participates in the regulation of ER stress-mediated apoptosis in osteoblasts, mouse osteoblastic MC3T3-E1 cells were treated with different doses of Dex (0, 10^−8^, 10^−6^, and 10^−4^ M) for 24 h. The expression of ATF6, phosphorylated PERK, and phosphorylated IRE1 were measured using western blot analysis. Compared with the control group, cells receiving Dex responded with dose-dependent increases in the levels of ATF6, phosphorylated PERK, and phosphorylated IRE1 (P < 0.05, Fig. 1a). Different doses of Dex may therefore induce ER stress in osteoblasts. TUNEL staining was used to confirm that Dex can induce ER stress-mediated osteoblast apoptosis. A dose of 10^−8^ M Dex had no effect on apoptosis in osteoblasts, compared to the control group (P > 0.05, Fig. 1b). Conversely, doses of 10^−6^ and 10^−4^ M Dex both significantly increased the rate of apoptosis in osteoblasts compared to that of the control (P < 0.05, Fig. 1b). Next, caspase-12 and caspase-3 activities were measured, and 10^−8^ M Dex had no effect on their activity compared to in the control (P > 0.05, Fig. 1c and d). Accordingly, 10^−6^ and 10^−4^ M Dex significantly increased the activity of caspase-12 and caspase-3 in osteoblasts, compared to the control (P < 0.05, Fig. 1c and d). These results suggest that different concentrations of Dex can induce ER stress in osteoblasts, with higher concentrations (10^−6^ and 10^−4^ M) inducing ER stress-mediated apoptosis in osteoblasts.

### High doses of Dex lead to ER stress-mediated apoptosis by inducing CHOP expression in osteoblasts

**Fig. 2:**
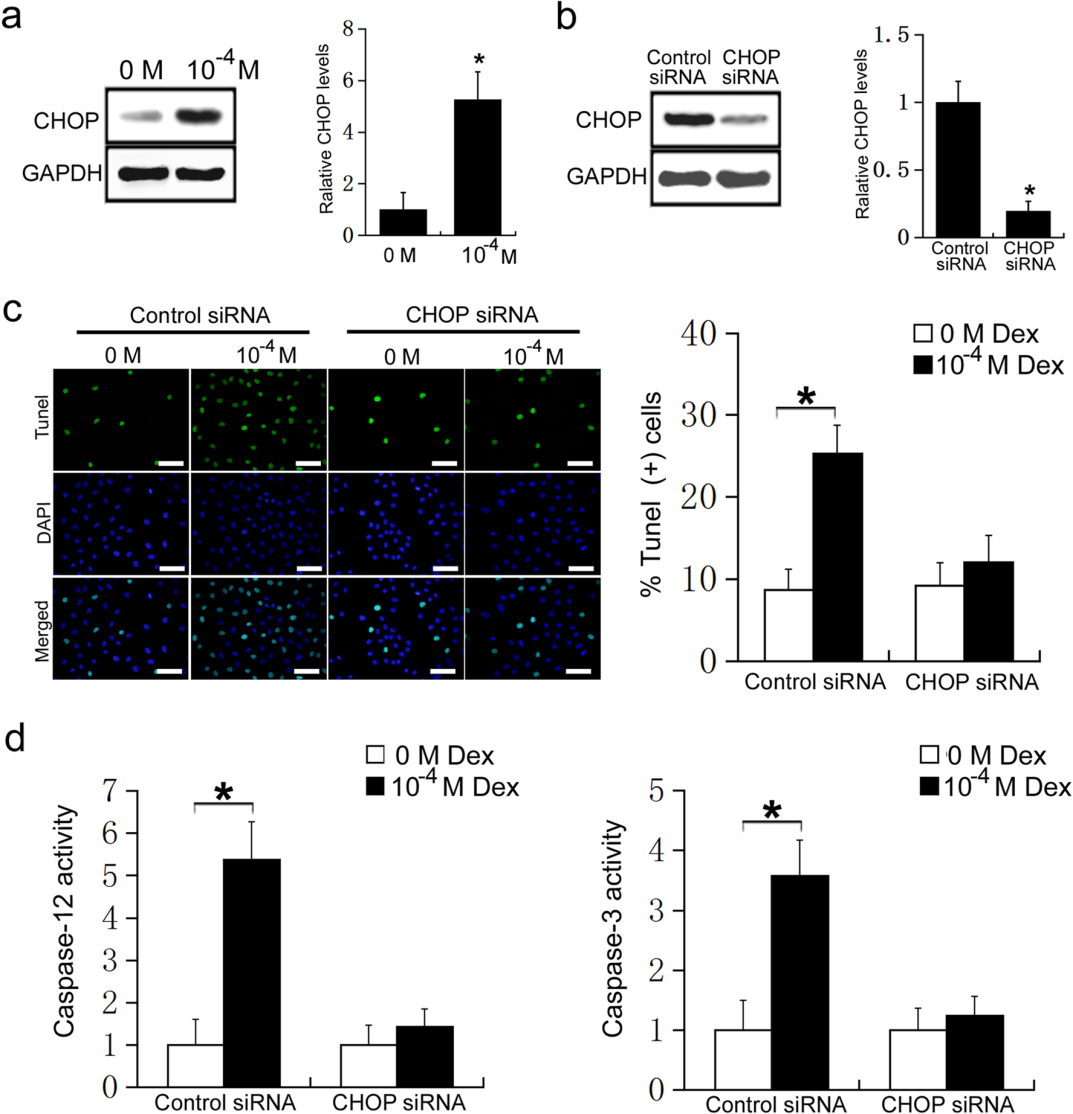
**a** MC3T3-E1 cells were treated with 0 or 10^−4^ M Dex, and the expression of CHOP was measured by western blot; **b** MC3T3-E1 cells were transfected with control siRNA or CHOP siRNA, and CHOP expression was detected by western blot. MC3T3-E1 cells were transfected with control siRNA or CHOP siRNA, and then treated with 0 or 10^−4^ M Dex for 24 h; **c** TUNEL staining was used to detect apoptosis in MC3T3-E1 cells (Scale=50 μm); **d** Caspase-12 activity and Caspase-3 activity

To further clarify the mechanism by which high doses of Dex regulate ER stress-mediated apoptosis in osteoblasts, MC3T3-E1 cells were treated with 0 or 10^−4^ M Dex, and CHOP expression was detected using western blot analysis. Compared with the control group, the expression of CHOP was significantly increased in cells receiving 10^−4^ M Dex (P < 0.05, Fig. 2a). To further confirm the effects of CHOP on Dex regulation of ER stress-mediated apoptosis in osteoblasts, CHOP siRNA was transfected into MC3T3-E1 cells. We found that CHOP expression was significantly decreased in MC3T3-E1 cells after transfection with CHOP siRNA (P < 0.05, Fig. 2b). MC3T3-E1 cells were transfected with CHOP siRNA or control siRNA, then treated with 0 or 10^−4^ M Dex. In the control siRNA group, 10^−4^ M Dex significantly increased the rate of apoptosis of MC3T3-E1 cells compared with 0 M Dex (P < 0.05, Fig. 2c). However, in the CHOP siRNA group, 10^−4^ M Dex did not change the rate of apoptosis of MC3T3-E1 cells compared with 0 M Dex (P > 0.05, Fig. 2c). Caspase-12 and caspase-3 activity assays further confirmed the results. In the control siRNA group, 10^−4^ M Dex significantly increased the activity of caspase-12 and caspase-3 compared with 0 M Dex (P < 0.05, Fig. 2d). However, in the CHOP siRNA group, 10^−4^ M Dex had no effect on caspase-12 and caspase-3 activity compared with 0 M Dex (P > 0.05, Fig. 2d). These results suggest that high doses of Dex induce ER stress-mediated apoptosis by promoting CHOP expression in osteoblasts. Moreover, CHOP expression is essential for high dose Dex-induced ER stress-mediated apoptosis in osteoblasts.

### High doses of Dex induce ER stress by promoting calcium influx in osteoblasts

**Fig. 3:**
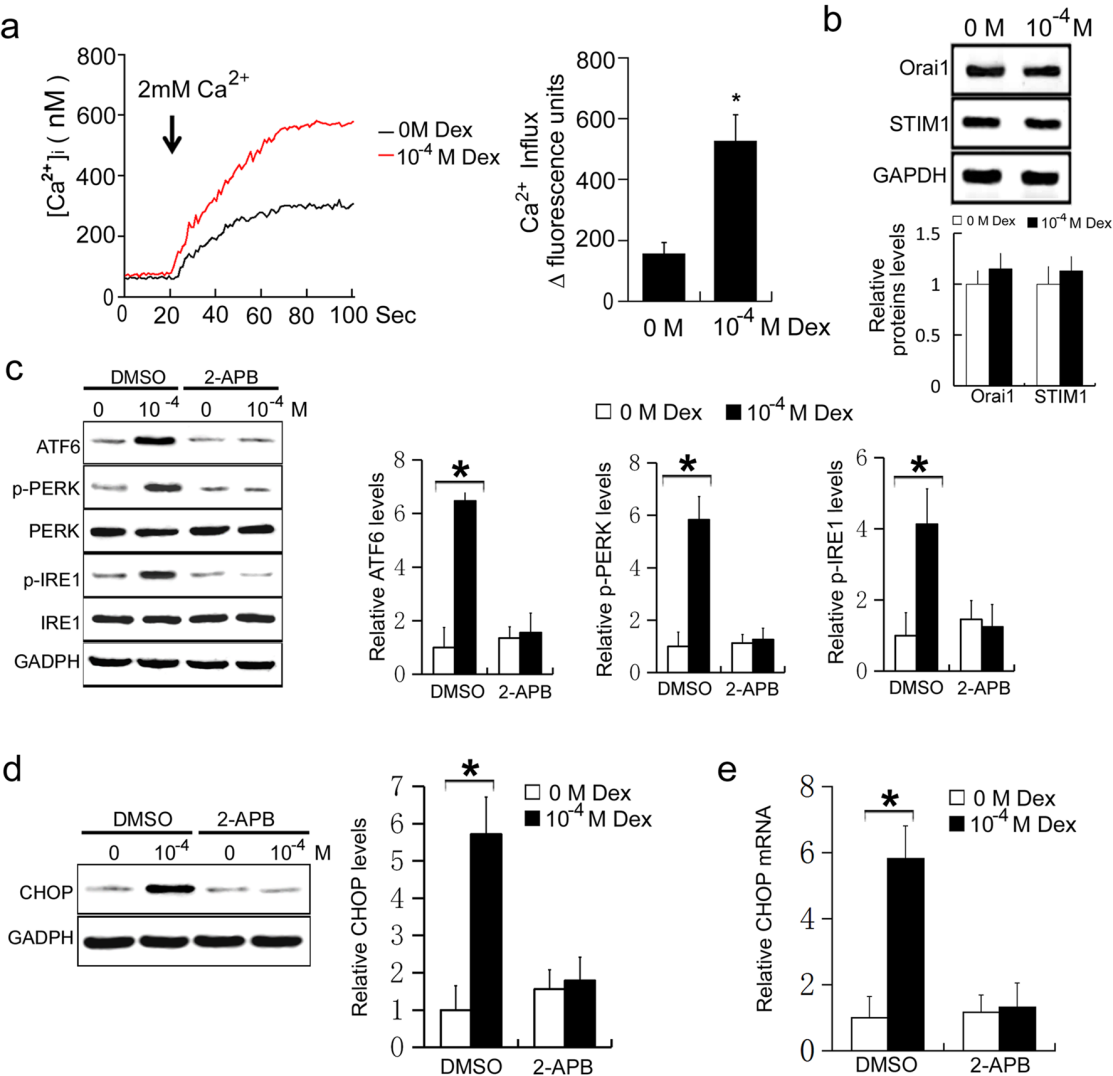
**a** MC3T3-E1 cells were treated with 0 or 10^−4^ M Dex for 24 h, and the calcium ion influx was detected by Fluo8-AM staining. **b** MC3T3-E1 cells were treated with 0 or 10^−4^ M Dex for 24 h, and the expressions of Orai1 and STIM1 were detected by Western blot. MC3T3-E1 cells were treated with DMSO or 2-APB, and then treated with 0 or 10^−4^ M Dex for 24 h. **c** Western blot of ATF6, phosphorylated PERK (p-PERK), PERK, phosphorylated IRE1 (p-IRE1), and IRE1 in MC3T3-E1 cells. **d** Western blot of CHOP in MC3T3-E1 cells. **e** Real-time PCR of CHOP mRNA in MC3T3-E1 cells

Calcium homeostasis imbalance induces ER stress [17–19]. To further clarify the mechanisms by which Dex induces ER stress in osteoblasts, MC3T3-E1 cells were treated with 0 or 10^−4^ M Dex, and the calcium ion influx was measured. Compared with the control group, calcium ion influx was significantly increased in osteoblasts receiving 10^−4^ M Dex (P < 0.05, Fig. 3a). This suggests that high doses of Dex promote calcium ion influx in MC3T3-E1 cells. To detect whether Dex affects the expression levels of STIM1 and Orai1, MC3T3-E1 cells were treated with 0 or 10^−4^ M Dex, and the expressions levels of STIM1 and Orai1 were measured using western blot. Compared with the control group, the expressions levels of STIM1 and Orai1 did not change in osteoblasts treated with 10^−4^ M Dex (P > 0.05, Fig. 3b). These results suggest that high doses of Dex have no effect on the expression of STIM1 and Orai1 in MC3T3-E1 cells. To further confirm the role of calcium ion influx in Dex-induced ER stress in osteoblasts, cells were simultaneously treated with Dex and 2-aminoethoxydiphenyl borate (2-APB), a blocker of store operated calcium influx. MC3T3-E1 cells were treated with 10 M 2-APB or DMSO, then treated with 0 or 10^−4^ M Dex. The expression levels of ATF6, phosphorylated PERK, and phosphorylated IRE1 were subsequently measured as before. Cells receiving DMSO and 10^−4^ M Dex had significantly increased levels of ATF6, phosphorylated PERK, and phosphorylated IRE1 compared with cells receiving DMSO and 0 M Dex (P < 0.05, Fig. 3b). However, in cells receiving 2-APB, 10^−4^ M Dex did not increase the levels of ATF6, phosphorylated PERK, or phosphorylated IRE1 compared with cells receiving 0 M Dex (P > 0.05, Fig. 3c). These results suggest that Dex induces ER stress by promoting calcium ion influx. The effect of calcium ion influx on ER stress was further verified by measuring the expression and mRNA transcription levels of CHOP. In the DMSO treatment group, 10^−4^ M Dex significantly increased the expression and mRNA levels of CHOP compared with 0 M Dex (P < 0.05, Fig. 3d and e). However, in the 2-APB treatment group, 10^−4^ M Dex did not increase the expression and mRNA levels of CHOP compared with 0 M Dex (P > 0.05 and P < 0.05, respectively, Fig. 3d and e). These results suggest that high doses of Dex induce ER stress by promoting calcium ion influx.

### 2-APB protects osteoblasts from Dex-induced ER stress-mediated apoptosis

**Fig. 4:**
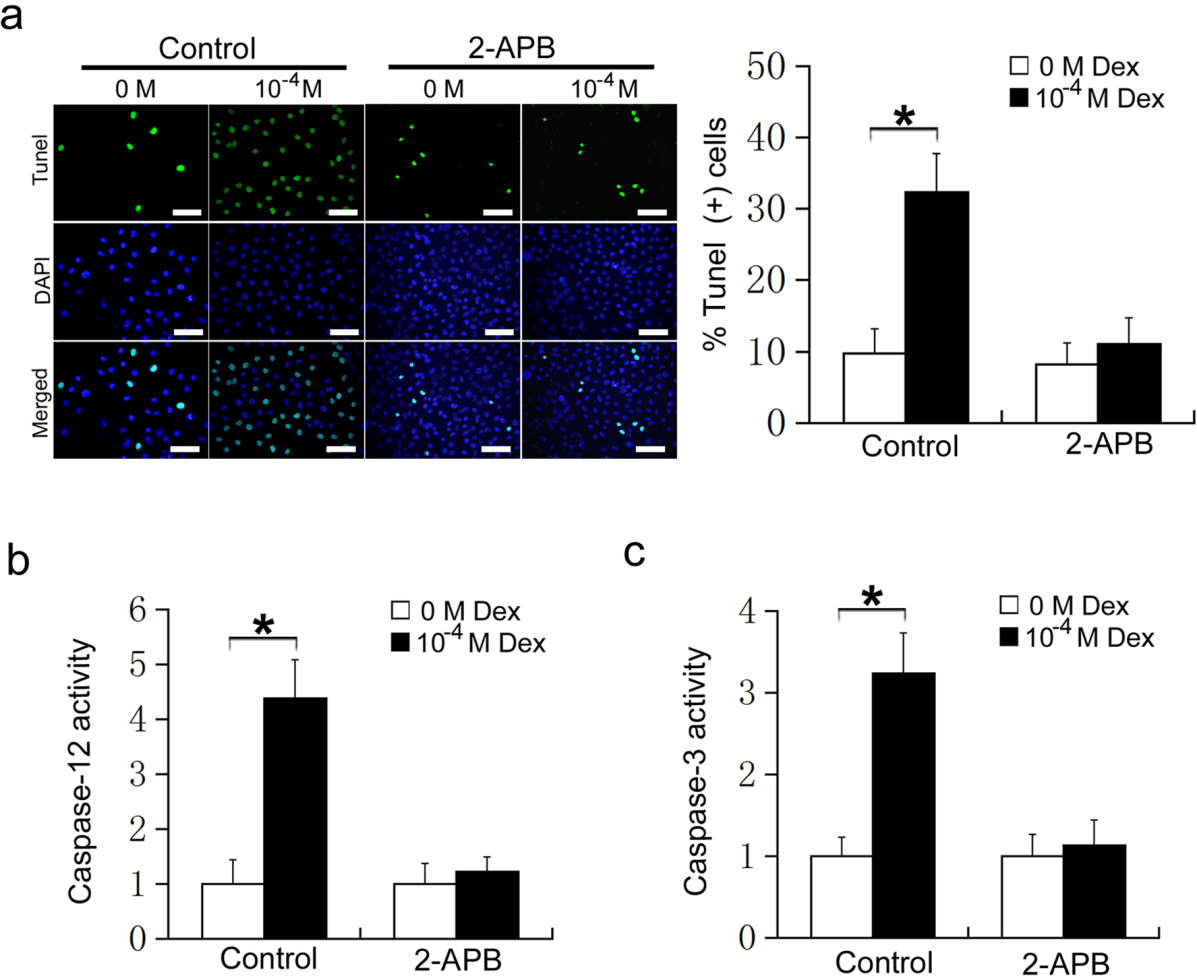
MC3T3-E1 cells were treated with DMSO or 2-APB, and then treated with 0 or 10^−4^ M Dex for 24 h. **a** TUNEL staining of MC3T3-E1 cells (Scale=50 μm) ; **b** Caspase-12 activity; **c** Caspase-3 activity

To further confirm the effect of 2-APB on Dex induction of ER stress-mediated apoptosis in osteoblasts, MC3T3-E1 cells were treated with 2-APB or DMSO, followed by 0 or 10^−4^ M Dex. Apoptosis was detected using TUNEL staining. In the DMSO group, 10^−4^ M Dex significantly increased the apoptosis of MC3T3-E1 cells compared with 0 M Dex (P < 0.05, Fig. 4a). However, in the 2-APB group, 10^−4^ M Dex did not increase the apoptosis of MC3T3-E1 cells compared with 0 M Dex (P > 0.05, Fig. 4a). Caspase-12 and caspase-3 activity assays further confirmed this result. In the DMSO treatment group, 10^−4^ M Dex significantly increased the activity of caspase-12 and caspase-3 compared with 0 M Dex (P < 0.05, Fig. 4b and c). However, in the 2-APB treatment group, 10^−4^ M Dex did not increase the activity of caspase-12 and caspase-3 compared with 0 M Dex (P > 0.05, Fig. 4b and c). These results suggest that 2-APB protects osteoblasts from Dex-induced ER stress-mediated apoptosis. Therefore, blocking calcium influx may be a new method to relieve Dex-induced osteoporosis.

## Discussion

Osteoporosis is the most serious adverse effect of Dex treatment [1–3]. The apoptosis of osteoblasts induced by Dex is the main cause of osteoporosis caused by Dex. Maintaining the correct number of osteoblasts is essential for bone formation. Decreases in the number of osteoblasts caused by excessive apoptosis plays a role in the pathogenesis of osteoporosis [4–6]. The response of cells to ER stress, including apoptosis, can have far-reaching effects in the body, and ER stress-mediated apoptosis is important in many diseases, including osteoporosis [21, 22].

Recent studies have shown that osteoblasts undergo ER stress during BMP-induced osteoblast differentiation. Some ER stress markers are also expressed and play an important role in osteoblast differentiation. OASIS is a key transcription factor of ER stress, which is involved in bone formation by promoting COL1A1 transcription and extracellular matrix secretion in osteoblasts [23]. XBP1, another important ER stress effector, can directly bind to the promoter region of Runx2, a key transcription factor for osteogenesis, thereby promoting osteogenesis [24]. ATF4, an important ER stress transcription factor, can promote collagen secretion and amino acid transport in osteoblasts [25, 26]. Although a large number of studies have confirmed that many key molecules of ER stress can promote osteogenesis, some have also found that excessive ER stress can lead to apoptosis of osteoblasts. Under excessive ER stress, the synthesis of ATF4 and ATF3 increases, promoting the expression of CHOP. High levels of CHOP expression activate the apoptotic pathway, leading to apoptosis of osteoblasts and accelerating the progression of osteoporosis [27–29].

A variety of injury factors acting on the ER can activate ER stress by causing intracellular calcium ion imbalances or luminal misfolding, leading to the accumulation of unfolded proteins [30]. There are three ER stress-sensing proteins residing in the endoplasmic omentum: activating transcription factor 6 (ATF6), RNA-activated protein kinase pancreatic elf-2 kinase (PERK), and inositol requiring enzyme 1 (IRE1). These proteins can bind GRP78/BIP to form a stable complex. In the absence of ER stress, these proteins are in a bound state and have no activity. When ER stress occurs, the accumulation of unfolded proteins allows GRP78/BIP to separate from the three transmembrane proteins. These three proteins are activated by dissociation from GRP78. IRE1 and PERK were activated by dimerization and autophosphorylation, respectively. ATF6 is transported from the endoplasmic omentum to the Golgi body, and then cleaved by a specific protease to form activated ATF6. Increased expression of ATF6, phosphorylated IRE1, and phosphorylated PERK is a marker of ER stress [31–33]. In the present study, the levels of ATF6, phosphorylated IRE1, and phosphorylated PERK in osteoblasts increased in a dose-dependent manner with 10^−8^, 10^−6^, and 10^−4^ M Dex, suggesting that Dex can induce ER stress in osteoblasts.

Caspases are the main effector molecules that mediate cell apoptosis. Caspase-12 is a specific molecule of ER stress-mediated apoptosis. Knockout of caspase-12 can significantly reduce ER stress-mediated apoptosis. Caspase-12 can mediate apoptosis through activation of cytochrome C-independent pathways, via caspase-9, caspase-7, and caspase-3 [34, 35]. Caspase-3 is a common molecule in many pathways that mediate apoptosis [36, 37]. In the present study, we found that 10^−8^ M Dex had no effect on apoptosis and the activation of caspase-12 and caspase-3, whereas 10^−6^ M and 10^−4^ M Dex significantly increased apoptosis and the activity of caspase-12 and caspase-3. High doses of Dex can therefore induce ER stress-mediated apoptosis in osteoblasts.

CHOP is a transcription factor that is specific to ER stress, which belongs to the transcription factor CCAAT/ enhancer binding protein (C/EBP) family, and often binds to other members of the transcription factor CCAAT/ enhancer binding protein (C/EBP) family, resulting in the formation of a dimer [38]. Under normal conditions, CHOP is expressed at low levels, but upon ER stress, activation of IRE1, PERK, and ATF-6 can promote the expression of CHOP. CHOP can induce apoptosis by promoting the expression of apoptotic genes, such as Bax, GADD34, and ERO1, but it can also induce apoptosis by inhibiting the expression of anti-apoptotic genes, such as Bcl-2 [39, 40]. In the present study, CHOP expression was significantly increased in osteoblasts after treatment with 10^−4^ M Dex. When CHOP expression was inhibited by CHOP siRNA, the Dex-mediated effects on apoptosis and caspase activity were abrogated. These results suggest that high doses of Dex can affect ER stress-mediated apoptosis by promoting CHOP expression in osteoblasts. Moreover, CHOP expression is essential for Dex-induced ER stress-mediated apoptosis in osteoblasts.

Calcium ions are important secondary messengers and play an important role in various cellular physiological and pathological activities. Calcium ion homeostasis is the basis for maintaining the normal structure and function of cells. The ER is the main site for protein synthesis, secretion, modification, and transport in cells, and also the main organelle for maintaining intracellular calcium ion homeostasis [41, 42]. The level of free calcium ions in the cytoplasm are much lower than in the ER under normal physiological conditions. The lyanodine receptor (RyR), the 1,4, 5-triphosphate receptor (IP3R), and the sarcoplasmic reticulum calcium pump are three calcium channels located in the ER, which are closely related to calcium ion release and calcium ion uptake. The dynamic homeostasis of intracellular calcium ions is maintained by the ER releasing calcium ions into the cytoplasm via RyR and IP3R, while calcium ions are taken up from the cytoplasm by calcium pumps into the ER [43, 44]. Exogenous stimulation can induce changes in calcium channels on the endoplasmic omentum, causing calcium depletion or overload, thus disrupting the synthesis, folding, and modification of proteins in the ER. Misfolded protein accumulation in the in the ER causes ER stress [45–47]. In the present study, calcium ion influx was significantly increased in osteoblasts receiving 10^−4^ M Dex, and 2-APB, a blocker of store operated calcium influx. Accordingly, 10^−4^ M Dex did not affect the levels of ATF6, phosphorylated PERK, and phosphorylated IRE1 after pretreatment with 2-APB. These results confirm that high doses of Dex induced ER stress by promoting calcium influx in osteoblasts. Similarly, 2-APB prevented the effects of Dex on CHOP expression, apoptosis, and the activation of caspase-12 and caspase-3. These results suggest that high doses of Dex induce ER stress by promoting calcium ion influx. The application of 2-APB protects osteoblasts from Dex-induced ER stress-mediated apoptosis. The well-known IP3 receptor (IP3R) antagonist, 2-APB, can inhibit the activation of store-operated Ca^2+^ entry (SOCE). SOCE is important for Ca^2+^ signaling and maintaining homeostasis in most mammalian cells and is mediated by STIM1 and Orai1. Reportedly, 2-APB does not affect the dimeric state of STIM1 but enhances the intramolecular coupling between the 1st predicted coiled-coil of STIM1 that holds STIM1 in an inactive conformation. Additionally, 2-APB promotes the STIM-Orai1-activating region of STIM1, with subsequent reduction in STIM1 puncta formation in the absence of Orai1, which helps prevent STIM1 activation. In our study, we found that high doses of Dex could promote calcium ion influx but had no effect on the expression of STIM1 and Orai1 in MC3T3-E1 cells [48, 49]. Therefore, we speculate that high doses of Dex affects the structural changes of STIM1 that eventually promote calcium ion influx. However, the specific mechanisms underlying Dex-mediated promotion of calcium ion influx warrants further studies.

## Conclusions

**Fig. 5:**
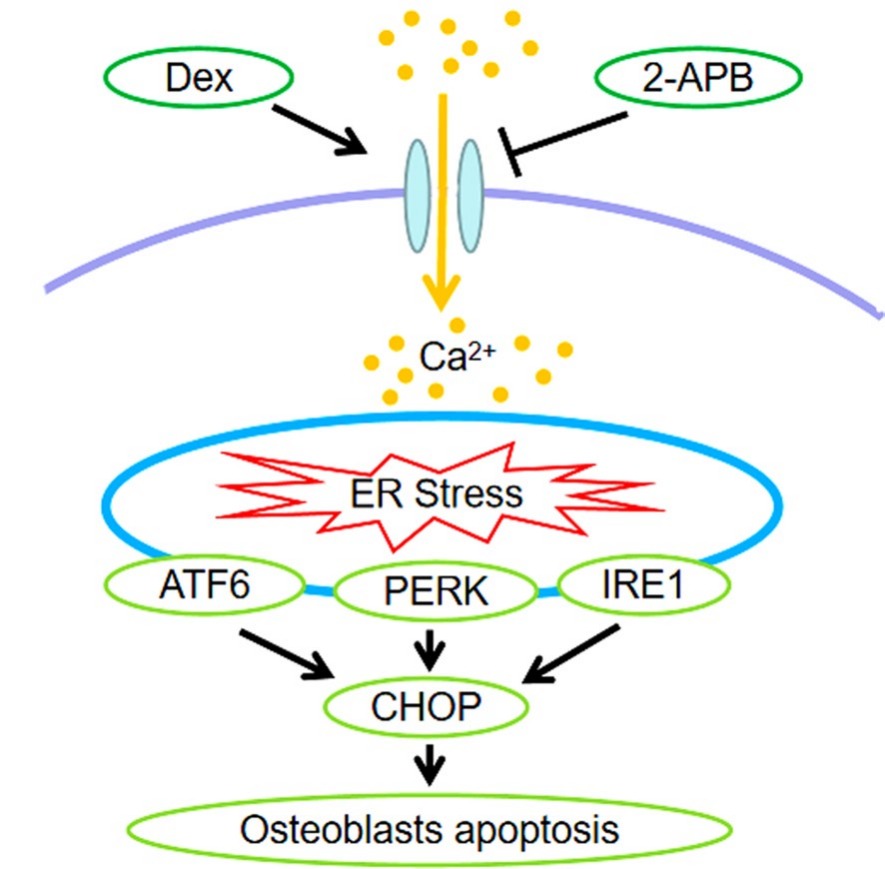
High doses of Dex induce CHOP expression by promoting calcium ion influx-dependent induction of ATF6, phosphorylated PERK and phosphorylated IRE1, which induce endoplasmic reticulum stress-mediated apoptosis in osteoblasts. 2-APB protects the osteoblasts from the effects of Dex, preventing endoplasmic reticulum stress-mediated apoptosis

In conclusion, in the present study we found that high doses of Dex induce CHOP expression by promoting calcium ion influx-dependent induction of ATF6, phosphorylated PERK and phosphorylated IRE1, which induce endoplasmic reticulum stress-mediated apoptosis in osteoblasts. 2-APB protects the osteoblasts from the effects of Dex, preventing endoplasmic reticulum stress-mediated apoptosis (Fig. 5). Therefore, blocking calcium influx may be a new avenue for treating Dex-induced osteoporosis. These findings provide a theoretical basis for developing novel methods for clinical prevention of osteoporosis induced by Dex.
